# Postgraduate education and training in drug development sciences: 30 years of the european center of pharmaceutical medicine

**DOI:** 10.3389/fphar.2024.1389349

**Published:** 2024-04-12

**Authors:** Annette Mollet, Nastazja Laskowski, Thomas Szucs

**Affiliations:** European Center of Pharmaceutical Medicine, University of Basel, Basel, Switzerland

**Keywords:** education, training, pharmaceutical industry, graduate programs, professional development courses, pharmaceutical medicine, medicine development, professional development

## Abstract

The European Center of Pharmaceutical Medicine (ECPM), affiliated with the University of Basel Department of Public Health, stands as a leading institution dedicated to advancing education in medicine development since 1991. At the heart of its educational offers lies the Diploma or Certificate (DAS or CAS) in Pharmaceutical Medicine, encompassing a comprehensive curriculum that covers the entire drug development process. ECPM has expanded its reach beyond Switzerland, offering courses in the USA, China and India. Through rigorous teaching and strategic alliances, ECPM continues to shape education in pharmaceutical medicine on an international scale.

## 1 Introduction

In the rapidly evolving field of medicines development, postgraduate education plays a vital role in equipping pharmaceutical industry professionals with up-to-date knowledge. This article explores the importance, structure and historical context of the courses available at the European Center of Pharmaceutical Medicine (ECPM) in Switzerland and its international partner institutes. We underscore the value of providing comprehensive training across critical areas such as discovery, clinical trials, regulatory activities and marketing in medicines development. These domains serve as the backbone of pharmaceutical medicine, and proficiency in these areas is of utmost importance. By fostering cooperation between stakeholders of the healthcare system, we have standardized and elevated education in medicines development. Anchored by the PharmaTrain syllabus, ECPM’s courses not only equip professionals with essential skills but also ensure they remain at the forefront of emerging trends and technologies.

## 2 About ECPM

### 2.1 Facts and figures–ECPM course offer

The European Center of Pharmaceutical Medicine (ECPM) (ECPM Office, 2024) or Institute of Pharmaceutical Medicine is dedicated to being the leading university institute for medicines and drug development in Europe. ECPM belongs to the Department of Public Health of the Medical Faculty at the University of Basel (University of Basel, 2024b; University of Basel, 2024a)) and operates with partners worldwide. The institute was founded in 1991 to cover the training needs of specialists working in drug development. It comprises an education and training department (est. 1991) and a research department (est. 2003). In 2009, the professorship in Pharmaceutical Medicine was inaugurated.

**FIGURE 1:**
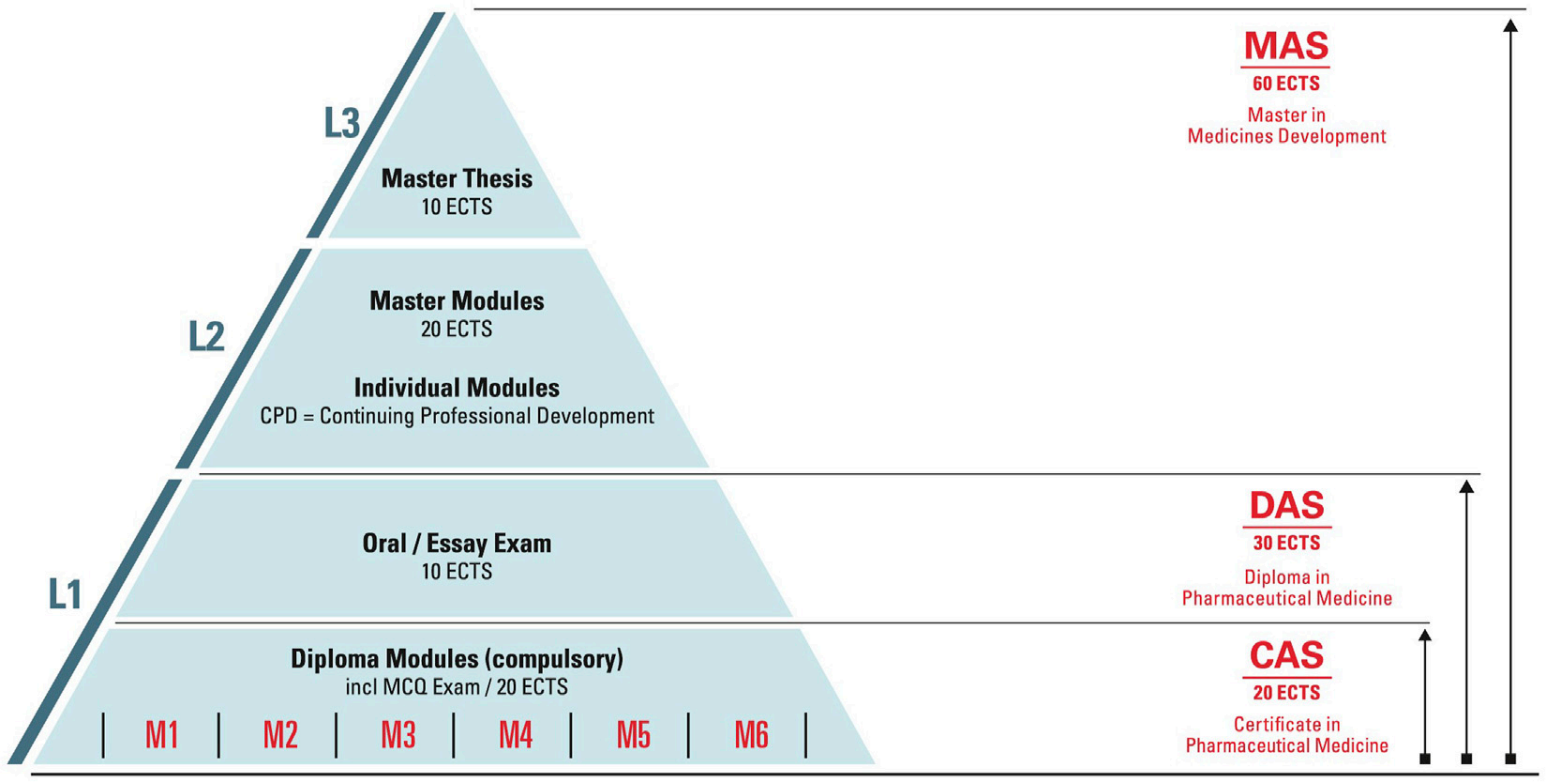
Postgraduate degree structure. In conformity with the Bologna system three postgraduate degrees are offered. These degrees can be achieved by completing the courses in a modular scheme, next to working full- or part-time and by acquiring the knowledge according to your needs. In addition, course participants who achieved a DAS can apply for a Swiss Specialist in Pharmaceutical Medicine title.

The education and training focus is on Advanced Studies, Postgraduate Training and Continuing Professional Development (CPD). The ECPM Diploma Course (ECPM Course) comprises six modules and represents the core of the postgraduate training platform (Figure 1). The course offers students to obtain either a Diploma (DAS) of Advanced Studies in Pharmaceutical Medicine or a Certificate of Advanced Studies (CAS) in Pharmaceutical Medicine and in addition a Master of Advanced Studies (MAS) in Medicines Development. The broad range of CPD short courses count towards the Master program, yet they are also a stand-alone offer and can be booked individually for continuing education. The curriculum provides a holistic understanding of the drug development process from molecule to market as well as key concepts in regulatory science, marketing and life cycle management. The program is based on the PharmaTrain Syllabus (pharmatrain, 2018). A cornerstone of the curriculum is the Frontiers in Drug Development seminar series, which introduces emerging trends and technologies in medicines development. This series plays a key role in ensuring that the participants and alumni stay at the forefront of the trends and development in pharmaceutical medicine.

**FIGURE 2:**
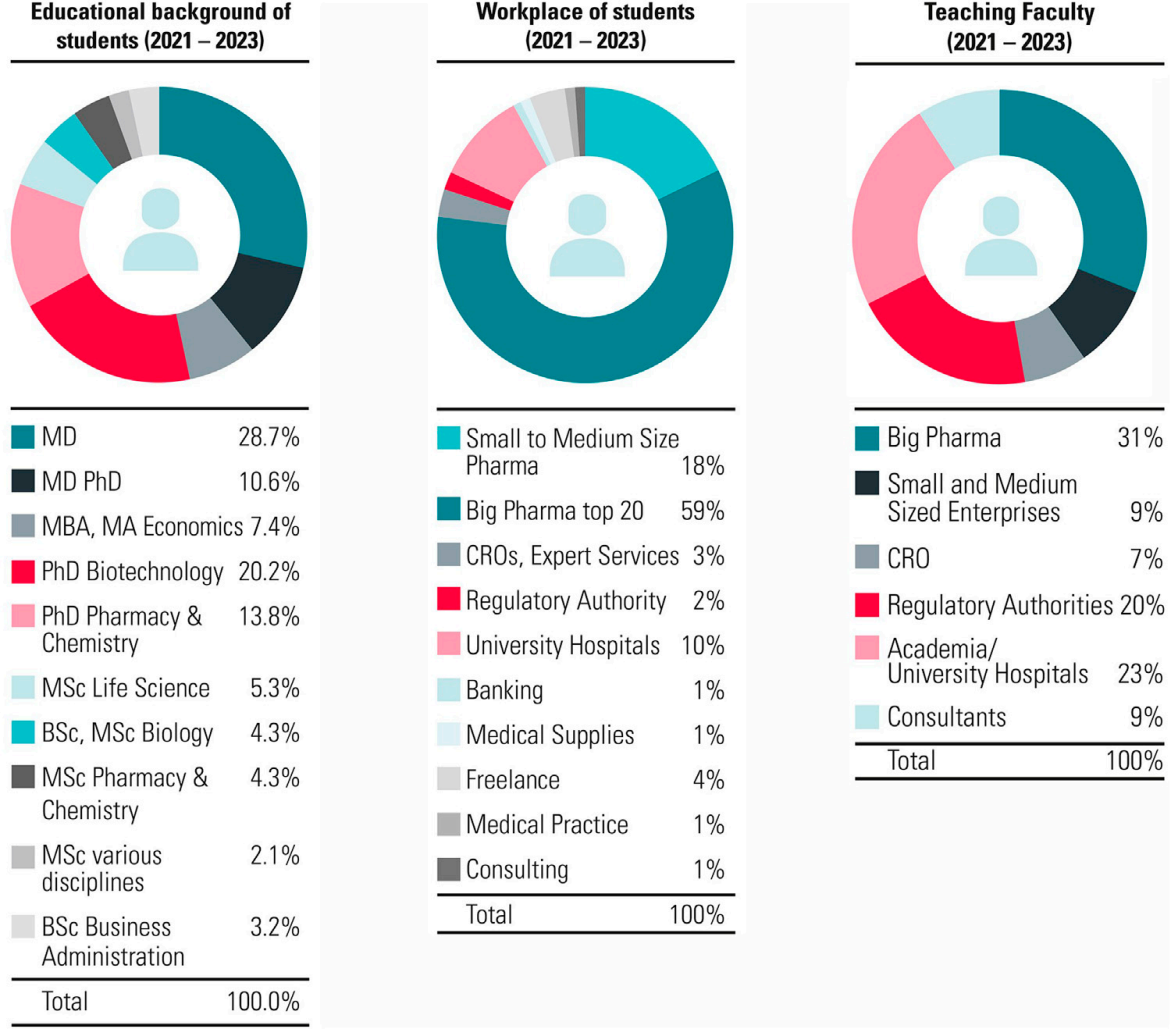
Characteristics of ECPM participants and faculty for the CAS/DAS. ECPM attracts participants and faculty mostly from across industry, academia and clinical settings. Some participants also join us from the banking and consulting sectors. The CAS/DAS course at ECPM provides an attractive insight for those interested in pharmaceutical medicine.

The mission at ECPM is to establish the best international training platform that provides and enhances the knowledge, expertise and skills required to perform modern discovery, development, regulation and marketing of medicinal products. Since the foundation in 1991, over 2,100 students holding MDs, PhDs or an MSc were trained and 150 members of faculty were recruited (Figure 2). Furthermore, since the introduction of the Bologna system in 2000, ECPM was proud to upgrade from awarding a certificate to the possibility of awarding a postgraduate diploma and later in 2015 a Master of Advanced Studies.

### 2.2 30 years at the forefront of drug discovery and development

The history of drug discovery and development goes back several thousand years. It started with the use of herbals in China and India. Later there was evidence of medicinal practice in Egypt. In Greece, Hippocrates started to transform medicine from art to science during the era of ancient medicine. The foundations of scientific medicine took over two thousand years to develop. Activities in Basel started in the second part of the 19th century coming out of the dye and chemical industry (Novartis, 2024; Roche Holding, 2024). Modern drug discovery started to emerge by the end of the 20th century with the arrival of organic chemistry, the discipline of pharmacology and germ theory. Later industrial technology made it possible to manufacture high quality medicines. By introducing genomics, the Human Genome Project (The national Institute of Health NIH, 2022) enabled advances in molecular and genomic medicine in the year 2000.

Tremendous progress occurred in the last 30 years, but we still have a limited understanding of disease pathology and progression. Cutting-edge technologies emerged, e.g., as humanized models in toxicology or gene sequencing. These help to predict and personalize the clinical success of drug candidates. Algorithms, machine learning, artificial intelligence and other *in silico* tools assist to study molecules in a dynamic state, even within a single cell. High-throughput technologies and digital devices produce an exponential amount of data. Hence it is important that such data is transformed to high quality information and subsequently turned into actionable knowledge. In all parts of this process, skills and talents of the individuals along the value chain are key. Our aim is to support research and educate professionals in drug discovery and development, especially at the interface of disciplines.

### 2.3 Highlights and milestones in the history of ECPM

**FIGURE 3:**
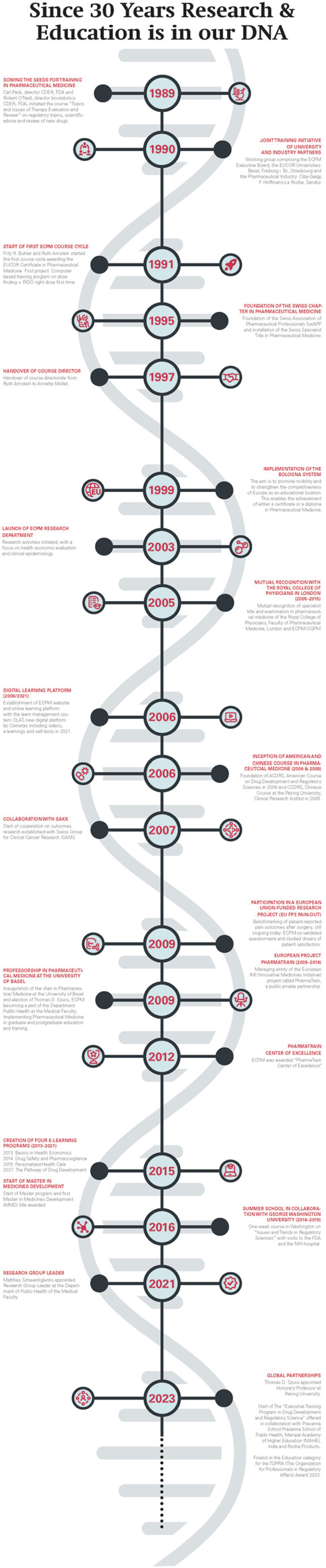
The historical DNA of ECPM. From 1989 to present ECPM has achieved many notable milestones in its development.

Robert O’Neill, Head of Biostatistics at FDA came to Basel in 1989 with the idea to develop a course in regulatory topics (Figure 3). The focus was to inform European scientists about how the U.S. Food and Drug Administration (FDA) (FDA, 2022) provides advice to sponsors of clinical studies and how FDA evaluates the evidence of the safety and efficacy of new drugs. Fritz R. Bühler, Professor in Cardiology and Pathophysiology and Head of the Department of Research at the University Hospital in Basel hosted Professor O’Neill during his sabbatical. Fritz R. Bühler had the idea to enhance the university–industry relationships by offering high quality education and training on a neutral, academic ground. The pilot course triggered a great response within academia and research-based pharmaceutical companies in Basel.

After the big success of the inaugural course, Fritz R. Bühler and Ruth Amstein, a pharmacist and PhD student of Fritz R. Bühler started the ECPM course in 1991 and developed a 2-year curriculum - the ECPM course was born. The course was part of the postgraduate training at the University of Basel within the medical faculty and partner of EUCOR (EUCOR, 2024), the European campus consisting of the Universities Basel, Freiburg im Breisgau and Strasbourg. Until 1999 the successful course led to an EUCOR certificate which, after the introduction of the Europe wide Bologna system (European Commission, 2024) in 2000, was upgraded to a postgraduate diploma. In 1997 Annette Mollet joined ECPM as the new course director. From 2009 to 2014, Fritz R. Bühler, Thomas D. Szucs, Susanne Daniel and Annette Mollet led the Innovative Medicines Initiative (IMI) (Innovative Medicines Initiative IMI, 2024; PharmaTrain, 2024) project–a public/private partnership called PharmaTrain. Through this European academic network, the ECPM training platform was taken to the next level to offer a postgraduate Master title. In 2012 ECPM was awarded PharmaTrain Center of Excellence and completed successfully two reassessments in 2018 and 2023 by PharmaTrain, reaffirming its prestigious status as a PharmaTrain Centre of Excellence for 12 consecutive years.

In 2003 the research group (ECPM Research, 2022) was established under the leadership of Matthias Schwenkglenks, who holds a PhD in Epidemiology and a Master in Public Health. Their research focus is on health economics, cost-benefit implications and the efficient use of pharmaceuticals and other healthcare interventions in Switzerland and internationally. Long-standing cooperation on outcomes research with the Swiss Group for Clinical Cancer Research (SAKK) and collaborations within the University Basel and Zurich and the pharmaceutical industry were established. One of the most important milestones was the establishment of a Professorship in pharmaceutical medicine and the election of Thomas D. Szucs at the University of Basel in 2009. Thanks to this ECPM became a full university institute within the department of public health of the medical faculty. The research department evolved over time and today consists of nine research scientists and a number of PhD and master students.

Since then, we are striving to implement teaching in drug development, health economics and policy also for undergraduate students and to continue to offer cutting edge courses by implementing digital course tools and offers. Thanks to our local and international collaboration with partners from the pharmaceutical industry, academia and governmental/regulatory authorities, our research and training remain an important support for modern drug development and informed and transparent health policy decision making.

In 2023, Matthias Schwenkglenks played a crucial role in the foundation of the new Basel Center for Health Economics (BCHE) (Basel, 2024), where he is co-leader. BCHE represents a collaborative effort between three departments in Universität Basel and Swiss TPH, led by Stefan Felder (Faculty of Economics), Günther Fink (Faculty of Economics, Swiss TPH) and Matthias Schwenkglenks (Faculty of Medicine). It will serve as a local and international networking hub, fostering connections between research, industry leaders, and policymakers.

### 2.4 A collaborative effort–the ECPM advisory board

Embodying the spirit of collaboration, the ECPM Advisory Board in Switzerland brings together members representing the pharmaceutical industry, academia, regulatory agencies and health insurance. Our unique combination of stakeholders creates a dynamic forum at the annual meetings, where inspiration and ideas for the development of ECPM flourish. We draw upon the experience of industry experts to understand unmet needs in pharmaceutical medicine, technological advancements, market trends and educational gaps in the workforce. The colleagues from academia contribute ideas for cutting-edge research and provide evidence-based perspectives. The involvement of regulatory agencies ensures that our collaborative efforts align with established standards, ethical considerations and comply with existing frameworks. Finally, the input of health insurance companies ensures an approach that equips our participants with the knowledge to work towards sustainable and cost-effective strategies in pharmaceutical medicine. Together, these pillars of expertise within the ECPM advisory board ensure strong foundations for ongoing innovation and excellence in education and training in pharmaceutical medicine.

### 2.5 The international footprint of ECPM–courses in the USA, China and India

Pharmaceutical companies, regulatory agencies, and governmental institutions act both locally and internationally as key stakeholders in the innovation and management of healthcare systems. To allow harmonization of education and training, ECPM has crossed borders and established roots in institutions outside of Switzerland. In 2006 and 2008, the University of Beijing and the subsidiary of the University of San Francisco in Washington adopted the course concept. In contrast to the format in Basel, where the course modules are offered over a period of 2 years, the American and Chinese courses cover 1 year.

In the US, the “American Course on Drug Development and Regulatory Science” ACRDS of the University of California San Francisco (American Course on Drug Development and Regulatory Science, 2024) was inaugurated in 2006 on their campus in Washington. This programme is strategically located with close proximity to the Food and Drug Association (FDA), with half of the course participants coming from the FDA itself. The other half of course participants represent industry, academia and service industry experts. This collaboration was initiated by Prof Carl Peck, the former head of the Center for Drug Evaluation and Research (CDER) and allows a great opportunity to exchange and to build up a professional network.

A comparable model is followed in Beijing, where the “Chinese Course on Drug Development and Regulatory Science” (CCDRS) offered by Peking University Clinical Research Institute (PUCRI) (Peking University Health Science Center, 2024) benefits from the establishment of a collaboration with the Chinese FDA (CFDA). It was with great pleasure that Thomas Szucs of ECPM and Carl Peck, co-founder of ECPM and ACDRS, received Honorary Professorships from Peking University on the 15-year anniversary of the Chinese Course of Drug Development and Regulatory Science (CCDRS) in 2023.

With excitement ECPM continues to expand its global footprint by establishing the “Executive Training Program in Drug Development and Regulatory Science” offered by ECPM, Centre for Regulatory Science, Department of Health Information, Prasanna School of Public Health (PSPH), Manipal Academy of Higher Education (MAHE) in India and the Roche India Healthcare Institute in 2022 (Manipal Academy of Higher Education, 2024; Roche India, 2024)). The course was initiated following a request from the former secretary general of EFPIA (European Federation of Pharmaceutical Industries and Associations) (European Federation of Pharmaceutical Industries and Associations, 2024) Richard Bergstrøm, and facilitated by Professors Angela and Helmut Brand, who hold professorships in public health both in Maastricht, the Netherlands and at PSPH, MAHE, India. Dr Lada Leyens was very instrumental in bridging the initiative since she was working at F. Hoffmann–La Roche in Basel and is holding an endowed chair at PSPH.

Due to the travel restrictions caused by the COVID pandemics, the course started in February 2023 online. The preparation, which was initiated much earlier, involved challenges in establishing contacts with key stakeholders in India. It was only once the Roche India Healthcare Institute came onboard, through the professional network of Dr Lada Leyens, that key connections could be established between industry partners, academia and the regulatory agency (Central Drugs Standard Control Organization, CDSCO). The training concept has been adapted to six 3-day modules over 1 year. Two days are dedicated to international topics and 1 day covers India-specific topics. Additional course content is offered as videos and case studies on the digital course platform. The inaugural course was done very interactively including group work to connect the participants online. The last module was offered on-site including visits to the Indian Council of Medical Research and the Indian Pharmacopeia and their laboratories. After this successful first cohort with 16 candidates, it became clear that the consortium needed to establish an advisory board with other pharmaceutical companies and public universities to make the course more known.

After its inaugural year, the Indian course was nominated for the Education category of the TOPRA (The Organization for Professionals in Regulatory Affairs) (TOPRA, 2024) Award 2023. The Education Award recognizes individuals who have made a significant contribution to the education and training of regulatory affairs, perhaps through the development of a formal educational qualification, the consistent delivery of a training product, or the production of an innovative in-house training programme.

### 2.6 Insights into the 30th anniversary of ECPM

ECPM was pleased to honor 30 years of excellence in continuous education, as described in detail in the annual report. Founded in 1991—as the first provider of postgraduate academic programs in pharmaceutical medicine both for medical doctors and a variety of academic experts working in drug development–over the past 30 years ECPM has established itself as one of the world’s leading university institutes for medicine and drug development, providing high-level education and training to more than 2,100 health professionals. On 20 June 2022—1 year later than foreseen due to COVID restrictions–the time had come for ECPM to celebrate its 30th anniversary with a formal ceremony and an exclusive anniversary seminar that spotlighted learnings and challenges in medicines development, followed by a look back on the highlights over the past 30 years of ECPM. To this end, we paid tribute to the legacy and landmark achievements of ECPM founder Prof. Fritz Bühler, who passed away in 2017. ECPM Co-founders Carl Peck and Robert O’Neill, formerly with the FDA, gave very personal memorial speeches. Several ECPM founding members and current board members honored ECPM’s accomplishments and milestones, with a number of distinguished representatives from the Pharmaceutical Industry, the Swiss Regulatory Authority, the Swiss Government, and the University of Basel.

### 2.7 Spotlight on anniversary celebration talks

At the core of the anniversary seminar was the presentation on the veritable DNA of ECPM by Thomas Szucs, current Director of ECPM. Further, Ruth Amstein, the former, and Annette Mollet, the current, Head of Education and Training, showed the development of ECPM marked by an impressive growth from a small associated institute to a full-fledged university institute of the Medical Faculty of the University of Basel. Another great highlight was the contribution of the directors of the partner universities San Francisco and Beijing who sent a highly inspiring video with a congratulatory message on the occasion of ECPM’s pearl anniversary. The attendees enjoyed a panel discussion of experts and alumni on the topic of “How to develop talents and competencies”—a highly informative and entertaining highlight that enriched the ECPM anniversary seminar. The discussion focused on the importance of the content taught in the ECPM training programs and the value of transferring it to the workplace and the daily challenges. The 30th anniversary was concluded with an evening ceremony, which started with of an exclusive classical concert with the Academic Orchestra Basel conducted by ECPM’s Director Professor Thomas Szucs. The historic event ended with an exquisite dinner and ample networking opportunities for course participants, guests, speakers, faculty, and members of the ECPM Advisory Board.

## 3 Conclusion

Over the course of 30 years, ECPM has learnt that in order to set up new training courses, it is crucial to establish relationships with the key players in a local healthcare system, getting to know their needs and challenges. Their support is not only important in defining the course content but also through sharing their expertise as speakers. Drug development and the successful functioning of a healthcare system are global aspects, which is why speakers also need to bring an international context and prepare our students for careers abroad. The creation of an international advisory board consisting of all key stakeholders is extremely important. On the one hand, this ensures the support of employers and, on the other hand, it gives us the opportunity to meet their needs precisely.

In order to succeed, you must stay updated on research and development, recognize trends and incorporate new technologies. Although we decided to return to on-site teaching after the COVID pandemic, digital technologies have allowed for great flexibility. Students can access not only all the course materials on our platform, but also videos of the individual lectures and additional background material. The cooperation and support from our sister courses abroad have been very important, especially during the pandemic, in order to maintain the courses on offer and to find new ways of communicating. This ensured that the quality of the training was not compromised.

From the inception of ECPM in 1991 to its current status as the largest provider of postgraduate education in medicines development, ECPM has continually evolved to provide participants with the most up-to-date knowledge at a high-quality level. The non-for-profit university status of ECPM allows us to bring together all stakeholders for discussion and exchange. Notable milestones, such as ECPM’s establishment as a full university institute within the Department of Public Health at the University of Basel and its recognition as a PharmaTrain Center of Excellence for 12 consecutive years, underscore its commitment to providing top-quality education. Its international footprint reflects its dedication to global collaboration and knowledge exchange. ECPM remains positioned to educate professionals worldwide to meet the challenges of tomorrow’s pharmaceutical medicine landscape.

## Data Availability

The original contributions presented in the study are included in the article/Supplementary material, further inquiries can be directed to the corresponding author.
